# Incidence, clinical features and risk factors of tacrolimus induced idiosyncratic liver injury in renal transplant recipients: A nested case-control study

**DOI:** 10.3389/fphar.2023.1126765

**Published:** 2023-03-13

**Authors:** Binbin Lv, Longshan Liu, Xiaoman Liu, Min Huang, Xiao Chen, Kejing Tang, Changxi Wang, Pan Chen

**Affiliations:** ^1^ Department of Pharmacy, The First Affiliated Hospital, Sun Yat-sen University, Guangzhou, China; ^2^ Institute of Clinical Pharmacology, School of Pharmaceutical Sciences, Sun Yat-sen University, Guangzhou, China; ^3^ Organ Transplant Center, The First Affiliated Hospital, Sun Yat-sen University, Guangzhou, China

**Keywords:** tacrolimus, drug induced liver injury, renal transplantation, incidence, risk factors, adverse drug reaction, hepatotoxicity

## Abstract

Rare data reported tacrolimus-induced liver injury (tac-DILI) in real world. We performed a nested case-control analysis of 1,010 renal transplant recipients. Recipients with tac-DILI were randomly matched at a ratio of 1:4 by the year of admission to the remaining recipients without tac-DILI to explore risk factors. The incidence of tac-DILI was 8.9% (95% CI = 7.2–10.7%). The most common type was cholestatic pattern (6.7%, 95% CI = 5.2–8.3%), followed by hepatocellular (1.6%, 95% CI = 0.8–2.4%) and mixed patterns (0.6%, 95% CI = 0.1–1.1%). 98.9% of recipients with tac-DILI have mild severity. The latency period were 42.0 (range, 21.5–99.8 days), 14.0 (range, 9.0–80.3 days), 16.0 (range, 11.5–24.5 days), and 49.0 days (range, 28.0–105.6 days) for total, hepatocellular, mixed, and cholestatic patterns, respectively. Baseline ALP level (OR = 1.015, 95% CI = 1.006–1.025, *p* = 0.002), age (OR = 0.971, 95% CI = 0.949–0.994*, p* = 0.006), and body weight (OR = 0.960, 95% CI = 0.940–0.982, *p* < 0.001) were independent risk factors. In conclusion, cholestatic pattern represents the most frequent type of tac-DILI. Young age, low body weight and abnormal baseline ALP level were risk factors.

## 1 Introduction

The prevalence of end-stage renal disease is increasing annually, and renal transplantation, rather than dialysis, is the optimal treatment option in terms of patient outcome. Standard maintenance immunosuppression regimens following renal transplantation commonly include calcineurin inhibitors (CNI), cyclosporine A or tacrolimus, mycophenolate mofetil (MMF), and corticosteroids (Bodell, 2015). Over the past few decades, tacrolimus has been the cornerstone of immunosuppressive therapy following transplants because it has proven to be effective in preventing acute rejection and maintaining graft function (Pirsch et al., 1997; Gonwa et al., 2003; Silva et al., 2007; Min et al., 2013). Moreover, tacrolimus is recommended as the first-line CNI in patients following renal transplantation by Kidney Disease: Improving Global Outcomes (KDIGO) clinical practice guidelines (Kasiske et al., 2010). However, the use of tacrolimus is associated with a variety of adverse reactions, such as nephrotoxicity, neurotoxicity, post-transplant diabetes mellitus, and hepatotoxicity, which may lead to poor prognosis in patients, with adverse symptoms ranging from milder ones such as headache and weight gain to more severe effects such as loss of renal function (Wijdicks, 2001; Nankivell et al., 2003; Rodriguez-Rodriguez et al., 2021).

To date, limited data have been published on tacrolimus-induced liver injury (tac-DILI), and the reference of tac-DILI is still limited to the drug label, for which the data originated from pre-marketing clinical trials. In the real-world, only few studies on tac-DILI have been reported. For example, one study reported that tacrolimus eventually resulted in cholestatic jaundice 60 days post-liver transplantation with increases in both alkaline phosphatase (ALP) and total bilirubin (TBIL) levels (Taniai et al., 2008). Another study showed that liver enzyme levels increased significantly 12 days post-renal transplantation, with aspartate aminotransferase (AST) elevated to 421 U/L, alanine aminotransferase (ALT) at 1242 U/L, and serum TBIL levels within the normal range (Mesar et al., 2013). Today, increasing accessibility to digital health data owing to the transition to electronic health records, together with the rising costs and known limitations of traditional clinical trials, real-world data offer enhanced research efficacy, which bridges the evidentiary gap between clinical research and practice (Corrigan-Curay et al., 2018).

Therefore, we aimed to investigate the clinical features of tac-DILI, including incidence, liver injury type, latency time, prognosis, and risk factors in renal transplant patients, in a real-world setting, which may serve as a reference for the diagnosis, prevention, and treatment of tac-DILI.

## 2 Methods

### 2.1 Study population and follow-up

This was a nested case-control study. Patients who had received renal transplantation between 1 January 2016, and 31 December 2021, were identified from the electronic medical records at the First Affiliated Hospital, Sun Yat-sen University. The electronic medical records contained detailed demographic, clinical, laboratory, imaging, and histologic (when available) data recorded both at presentation and at follow-up evaluation of the patients. The inclusion criteria were as follows: 1) age (no bar) and 2) receiving tacrolimus as a maintenance immunosuppressive agent for the first-time post-transplantation. The exclusion criteria were as follows: 1) secondary renal transplantation; 2) incomplete laboratory data (lack of data obtained within 7 days prior to tacrolimus medication or lack of follow-up liver function tests); 3) patients receiving tacrolimus therapy prior to admission without baseline data; 4) abnormal liver enzyme levels that reached the liver injury standards prior to medication or within 3 days post-surgery; 5) patients with malignancy, hepatic hemangioma, active hepatitis, or other underlying liver disease at admission. The study was approved by the Ethics Committee for Clinical Research and Animal Trials of the First Affiliated Hospital of Sun Yat-sen University (No. 2021199). This study was carried out in accordance with the requirements of the Declaration of Helsinki. No donor organs had been obtained from executed prisoners and that organs were procured after informed consent or authorization.

### 2.2 Case-control selection

Patients with tac-DILI were identified using the Roussel Uclaf causality assessment method (RUCAM). The date on which the liver met the criteria for injury was defined as the index date. We chose a risk-set sampling approach that patients with tac-DILI were randomly matched with the remaining patients without tac-DILI at a ratio of 1:4 by year of admission to identify risk factors. For each new tac-DILI case, a control patient was randomly extracted from the risk-set cohort in the same year. Random matching method adopted extraction from a random number table.

### 2.3 Diagnostic criteria for tac-DILI

According to the European association for the study of the liver (EASL) clinical practice guidelines (CPG) of DILI(EASL, 2019), patients meeting the following criteria were defined as suspected DILI cases: 1) ALT ≥5× upper limit of normal (ULN); 2) ALP ≥2× ULN, particularly with accompanying elevations in concentrations of gamma-glutamyl transferase (GGT) in the absence of known bone pathology driving the rise in ALP level; and 3) ALT ≥3× ULN and TBIL ≥2× ULN. For patients with abnormal liver enzyme levels prior to tacrolimus treatment, the ULN was replaced by the baseline values, and elevations were calculated proportionate to the modified baseline.

For patients who met the criteria for liver injury, we examined alternative causes of hepatitis in detail, including hepatitis A virus, hepatitis B virus, hepatitis C virus, Epstein-Barr virus or cytomegalovirus based on serological evidence and biliary stricture, hepatic artery thrombosis, portal/hepatic venous stenosis or thrombosis based on imaging evidence. Furthermore, we also assessed the possibility of concomitant agents that may contribute to DILI, including dose, the dates of the start and discontinuation of therapy, the date of onset of the first abnormal laboratory test result, the initial laboratory results at presentation and the liver histology results.

### 2.4 Causality assessment of tac-DILI

All patients with alternative causes of liver injury were excluded. The others were assessed using the RUCAM scale, and the evaluation criteria were as follows: 1) 1–2, unlikely; 2) 3–5, possible; 3) 6–8, probable; and 4) ≥ 8, highly probable. After the independent causality assessment, the suspected tac-DILI cases were reviewed carefully between two pharmacists and one clinician to ensure agreement (by consensus) on all assessments. Patients with a score of ≥3 were included in the DILI group.

### 2.5 Classification of tac-DILI patterns and severity

According to the EASL CPG of DILI, patients with DILI were categorized into three patterns based on the ratio R): 1) hepatocellular pattern: ALT alone is elevated ≥ 5-fold above the ULN or R ≥ 5; 2) cholestatic pattern: ALP alone is elevated ≥ 2-fold above the ULN or R ≤ 2; 3) mixed pattern: R > 2 to <5. The R value was calculated on the day when the peak liver enzyme value met the DILI standard (R = [ALT present/ALT baseline]/[ALP present/ALP baseline]).

The severity of DILI is classified into four grades: 1) grade 1 (mild): ALT ≥5 or ALP ≥2 and TBL <2 ULN; 2) grade 2 (moderate): ALT ≥5 or ALP ≥2 and TBL ≥2 ULN, or symptomatic hepatitis; 3) grade 3 (severe): ALT ≥5 or ALP ≥2 and TBL ≥2 ULN, or symptomatic hepatitis and one of the following criteria: a) INR ≥1.5, b) ascites and/or encephalopathy, disease duration <26 weeks, and absence of underlying cirrhosis, or c) other organ failure due to DILI; and 4) grade 4 (fatal/transplantation): death or liver transplantation due to DILI.

### 2.6 Outcomes of tac-DILI

We followed the prognosis of patients with tac-DILI, including recovery, improvement, no improvement, and aggravation. “Recovery” was defined as a decrease in liver enzyme levels to the ULN or baseline value, ‘improvement’ was defined as a decrease in liver enzyme levels below the criteria of liver injury or baseline value, “no improvement” was defined as related liver enzyme levels not decreasing below the criteria of liver injury or baseline value, and “aggravation” was defined as liver enzyme levels beyond the peak value.

### 2.7 Statistical analysis

Continuous variables were summarized as medians (interquartile ranges, IQR). Categorical variables were presented as numbers and proportions. Comparisons of baseline demographic and clinical characteristics between cases and controls were performed using the *χ* ^2^ test for categorical variables and the independent *t*-test or Mann-Whitney *U* test for continuous variables. Variables with *p* < 0.1 in the univariate analysis were analyzed using the logistic regression model. A backward stepwise logistic regression model was used to analyze independent risk factors for tac-DILI. Associations were expressed as odds ratios (ORs) with 95% confidence intervals (CIs). Statistical significance was set at *p* < 0.05. Data analysis was performed using IBM SPSS statistics version 25 (SPSS Inc.) and GraphPad Prism 8 (GraphPad Software).

## 3 Results

### 3.1 Study population

**FIGURE 1:**
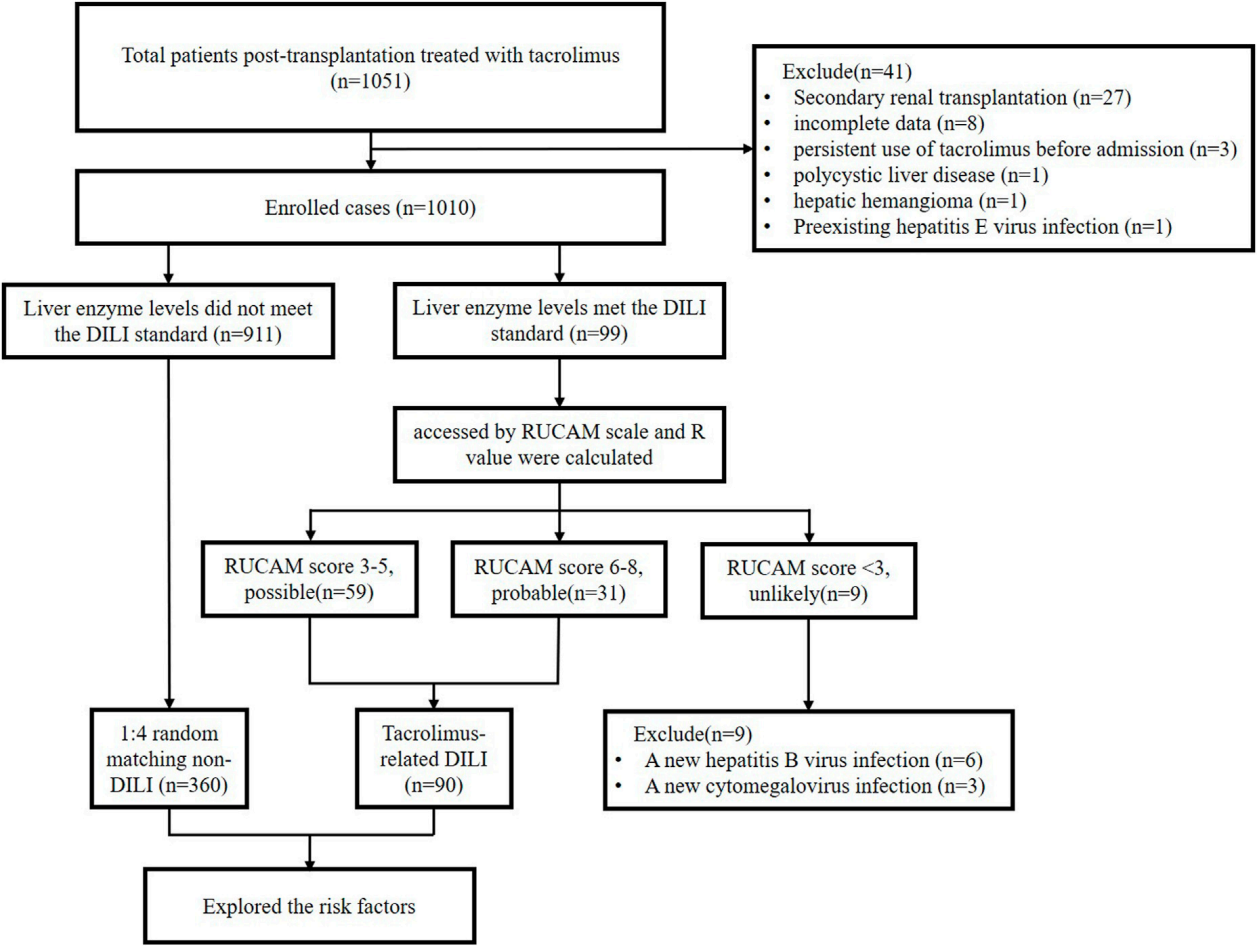
Study flow diagram.

A total of 1,051 post-renal transplant recipients were enrolled in the study. Among these, 41 recipients were excluded based on the exclusion criteria. Of the 1,010 recipients enrolled, 99 met the liver injury criteria and were assessed using RUCAM (Figure 1).

### 3.2 Clinical characteristics, severity, and outcome of patients with tac-DILI

**FIGURE 2:**
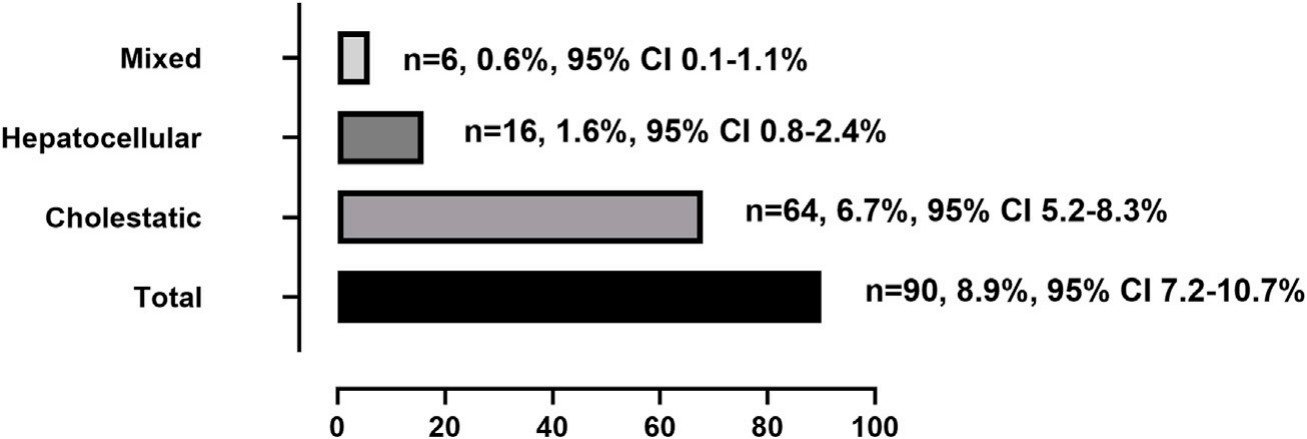
Incidence of total and different patterns of tacrolimus-induced liver injury.

The clinical characteristics of patients with tac-DILI are shown in Table 1 tac-DILI was confirmed in 90 patients, with an incidence of 8.9% (95% CI = 7.2–10.7%). A total of 59 patients with RUCAM scores of 3–5 was regarded as ‘possible’, and 31 patients with RUCAM scores of 6–8 were regarded as ‘probable’. The incidence of the different tac-DILI patterns is shown in Figure 2. These cases were evaluated and classified according to their R values at the time of liver injury. Among them, 16 presented with hepatocellular pattern, 6 with mixed pattern, and 68 with cholestatic pattern. A total of 89 tac-DILI cases showed mild and one case showed moderate severity.

**TABLE 1:** Characteristics and outcomes of tacrolimus-induced liver injury.

Variables		Total (*n* = 90)	Pattern of liver injury		
			Hepatocellular (=16)	Mixed (*n* = 6)	Cholestatic (*n* = 68)
Causality assessment					
	possible, n (%)	59 (65.6%)	11 (68.8%)	3 (50.0%)	45 (66.2%)
	probable, n (%)	31 (34.4%)	5 (31.2%)	3 (50.0%)	23 (33.8%)
Severity					
	Mild	89 (98.9%)	16 (100%)	6 (100%)	67 (98.5%)
	Moderate	1 (1.1%)	0 (0.0%)	0 (0.0%)	1 (1.5%)
	Severe	0 (0.0%)	0 (0.0%)	0 (0.0%)	0 (0.0%)
	Fatal/transplantation	0 (0.0%)	0 (0.0%)	0 (0.0%)	0 (0.0%)
Latency time of abnormal liver enzyme, median (IQR), days		15.0 (9.0–18.0)	10.0 (5.0–63.8)	15.0 (7.5–23.0)	15.5 (9.0–26.0)
Latency time of liver injury, median (IQR), days		42.0 (21.5–99.8)	14.0 (9.0–80.3)	16.0 (11.5–24.5)	49.0 (28.0–105.6)
Adjustment measure of tacrolimus					
	Withdrawal/change	3 (3.3%)	0 (0.0%)	0 (0.0%)	3 (4.3%)
	Dose reduction	79 (87.8%)	15 (93.8%)	3 (75.0%)	61 (87.1%)
	Without dose adjustment	8 (8.9%)	1 (6.2%)	1 (25.0%)	6 (8.6%)
Concomitant immunosuppressive medication during liver injury					
	Glucocorticoids	84 (93.3%)	15 (93.8%)	6 (100%)	63 (92.6%)
	Mycophenolate Mofetil/Mycophenolate Sodium	88 (97.8%)	15 (93.8%)	6 (100%)	67 (98.5%)
	Mizoribine	2 (2.2%)	1 (6.2%)	0 (0.0%)	1 (1.5%)
C_0_ of tarolimus during liver injury, median (IQR), μg/L		6.8 (5.9–9.6)	8.0 (6.3–10.5)	9.9 (6.55–16.95)	6.7 (5.9–9.1)
	<6 μg/L, n (%)	23 (25.6%)	3 (18.75%)	1 (16.7%)	19 (30.0%)
	6–12 μg/L, n (%)	55 (61.1%)	12 (75%)	3 (50.0%)	40 (58.8%)
	>12 μg/L, n (%)	5 (13.3%)	1 (6.25%)	2 (33.3%)	9 (13.2%)
hepatoprotective agents					
	Anti-inflammatory agents	20 (22.2%)	8 (50.0%)	1 (16.7%)	11 (16.2%)
	Liver membrane protectors	19 (21.1%)	9 (56.3%)	4 (66.7%)	6 (8.8%)
	Antioxidants	29 (32.2%)	9 (56.3%)	5 (83.3%)	15 (22.1%)
	Cholagogue	6 (6.7%)	2 (12.5%)	1 (16.7%)	3 (4.4%)
	Antidotes	8 (8.9%)	4 (25.0%)	2 (33.3%)	2 (2.9%)
Outcome of liver injury					
	Recovery	34 (37.8%)	12 (75.0%)	4 (66.7%)	18 (26.5%)
	Improvement	35 (38.9%)	4 (25.0%)	2 (33.3%)	29 (42.6%)
	No improvement	15 (16.7%)	0 (0.0%)	0 (0.0%)	15 (22.1%)
	Deterioration	6 (6.7%)	0 (0.0%)	0 (0.0%)	6 (8.8%)
Time to recovery, median (IQR), days		65.0 (16.8–397.8)	22.0 (12.3–28.8)	8.0 (4.5–70.0)	390.5 (199.0–604.3)

Latency time indicates both the time from the initial dosing to the onset of liver enzyme abnormality and meeting the criteria of liver injury in patients with tac-DILI. The median latency time of liver enzyme abnormality was 15.0 (range, 9.0–18.0 days), 10.0 (range, 5.0–63.8 days), 15.0 (range, 7.5–23.0 days) and 15.5 days (range, 9.0–26.0 days) for total, hepatocellular, mixed and cholestatic patterns, respectively. The median latency time to meet the criteria of liver injury was 42.0 (range, 21.5–99.8 days), 14.0 (range, 9.0–80.3 days), 16.0 (range, 11.5–24.5 days) and 49.0 days (range, 28.0–105.6 days) for total, hepatocellular, mixed and cholestatic patterns, respectively (Table 1). Only three patients (with cholestatic pattern) switched to cyclosporine A due to tacrolimus intolerance. Dose reduction was performed in 79 tac-DILI cases, including 15 cases with hepatocellular pattern, three case with mixed pattern, and 61 cases with cholestatic pattern. Only eight patients continued tacrolimus treatment without dose adjustment following tac-DILI: one with hepatocellular pattern, one with mixed pattern, and six with cholestatic pattern. For co-medication during liver injury, 84 patients simultaneously received glucocorticoids and 88 received MMF.

The whole blood trough concentration (C_0_) of tacrolimus was measured in patients with tac-DILI on the day of liver injury, and the median value of C_0_ for the total population was 6.8 μg/L (range, 5.9–9.6 μg/L). However, when we compared the C_0_ values among the different patterns of liver injury, no significant differences were found. Furthermore, there was also no significant difference in C_0_ of tacrolimus between patients with and without tac-DILI (Table 2).

**TABLE 2:** Characteristics between patients with and without tacrolimus-induced liver injury.

Characteristics		DILI group (*n* = 90)	Non-DILI group (*n* = 360)	*p*-Value
Age, median (IQR), year		16.0 (9.0–37.3)	39.0 (30.0–49.8)	**0.000**
	≤18 years, n (%)	48 (53.3%)	28 (7.8%)	**0.000**
	>18 years, n (%)	42 (46.7%)	332 (92.2%)	
Sex, n (%)				
	Male	46 (51.1%)	233 (64.7%)	**0.017**
	Female	44 (48.9%)	127 (35.3%)	
Weight, median (IQR), kg		41.5 (21.6–55.2)	58.5 (50.0–60.0)	**0.000**
BMI, median (IQR), kg/m^2^		17.6 (14.3–20.9)	21.5 (19.0–13.7)	**0.000**
	<18.5 kg/m^2^	50 (55.6%)	34 (9.4%)	**0.000**
	18.5–24 kg/m^2^	30 (33.3%)	114 (31.7%)	
	>24 kg/m^2^	10 (11.1%)	32 (8.9%)	
Ethnic groups, n (%)				0.542
	Han	87 (96.7%)	352 (97.8%)	
	Other ethnic minorities	3 (3.3%)	8 (2.2%)	
Blood type, n (%)				0.373
	A	31 (34.4%)	91 (25.3%)	
	AB	8 (8.9%)	36 (10.0%)	
	B	22 (24.4%)	105 (29.2%)	
	O	29 (32.2%)	128 (35.6%)	
Payment method, n (%)				0.405
	Medical insurance	78 (86.7%)	323 (89.7%)	
	Self-paying	12 (13.3%)	37 (10.3%)	
Underlying disease, n (%)				
	Diabetes mellitus	4 (4.4%)	33 (9.2%)	0.145
	Hypertension	71 (78.9%)	305 (84.7)	0.182
Biochemical parameters baseline values				
	ALT [1–40 U/L], median (IQR), U/L	20.0 (10.1–26.0)	19.0 (12.0–26.0)	0.681
	AST [1–37 U/L], median (IQR), U/L	24.0 (17.5–31.0)	18.0 (13.0–23.0)	**0.000**
	ALP [0–110 U/L], median (IQR), U/L	94.0 (80.0–105.3)	71.0 (56.0–90.0)	**0.000**
	ALB [35–50 g/L], median (IQR), g/L	42.0 (38.0–45.4)	42.7 (38.4–47.0)	0.111
	TBIL [3.0–22.0 μmol/L], median (IQR), μmol/L	9.0 (6.9–12.2)	9.7 (8.0–11.8)	0.116
	CREA [53–115 μmol/L], median (IQR), μmol/L	797.5 (590.0–990.3)	861.5 (688.0–1,096.5)	**0.008**
	WBC [4.00-10.00 × 10^9/L], median (IQR), 10^9/L	8.2 (6.3–9.8)	7.5 (5.8–9.3)	0.052
	RBC [4.00-5.50 × 10^9/L], median (IQR), 10^9/L	3.5 (3.0–4.2)	3.6 (3.1–4.1)	0.357
	Hemoglobin [120–140 g/L], median (IQR), g/L	102.0 (87.0–121.3)	105.0 (91.0–120.0)	0.451
	Hematocrit [42.0-49.0], median (IQR), %	30.2 (26.8–35.2)	31.6 (27.4–35.0)	0.305
	Platelet [100–300 g/L], median (IQR), g/L	208.0 (165.8–259.0)	176.5 (143.0–222.0)	**0.001**
	INR [0.80-1.15]	0.99 (0.94–1.1)	0.99 (0.94–1.05)	0.926
C_0_ of tacrolimus at the first discharge, median (IQR), μg/L		7.6 (6.4–9.7)	7.9 (6.6–9.6)	0.545

Bold values represent *p*<0.05.

Notably, 45 of the 90 patients with tac-DILI received hepatoprotective agents, with antioxidants being used most frequently, followed by anti-inflammatory agents, liver membrane protectors, cholagogues, and antidotes (Table 1). Regarding the outcomes of patients with tac-DILI, 34 recovered and 35 showed improvement, but 15 patients showed no improvement and six patients showed deterioration. In addition, patients with hepatocellular pattern showed the highest rate of recovery (75.0%) and outcomes except recovery were found to be higher in patients with cholestatic pattern (46% improvement, 22.1% no improvement, and 8.8% deterioration). The median time to recovery was 65.0 (range, 16.8–397.8 days), 22.0 (range, 12.3–28.8 days), 8.0 (range, 4.5–70.0 days), and 390.5 days (range, 199.0–604.3 days) for total, hepatocellular, mixed and cholestatic pattern, respectively.

### 3.3 Changes in biochemical parameters associated with tacrolimus treatment

**FIGURE 3:**
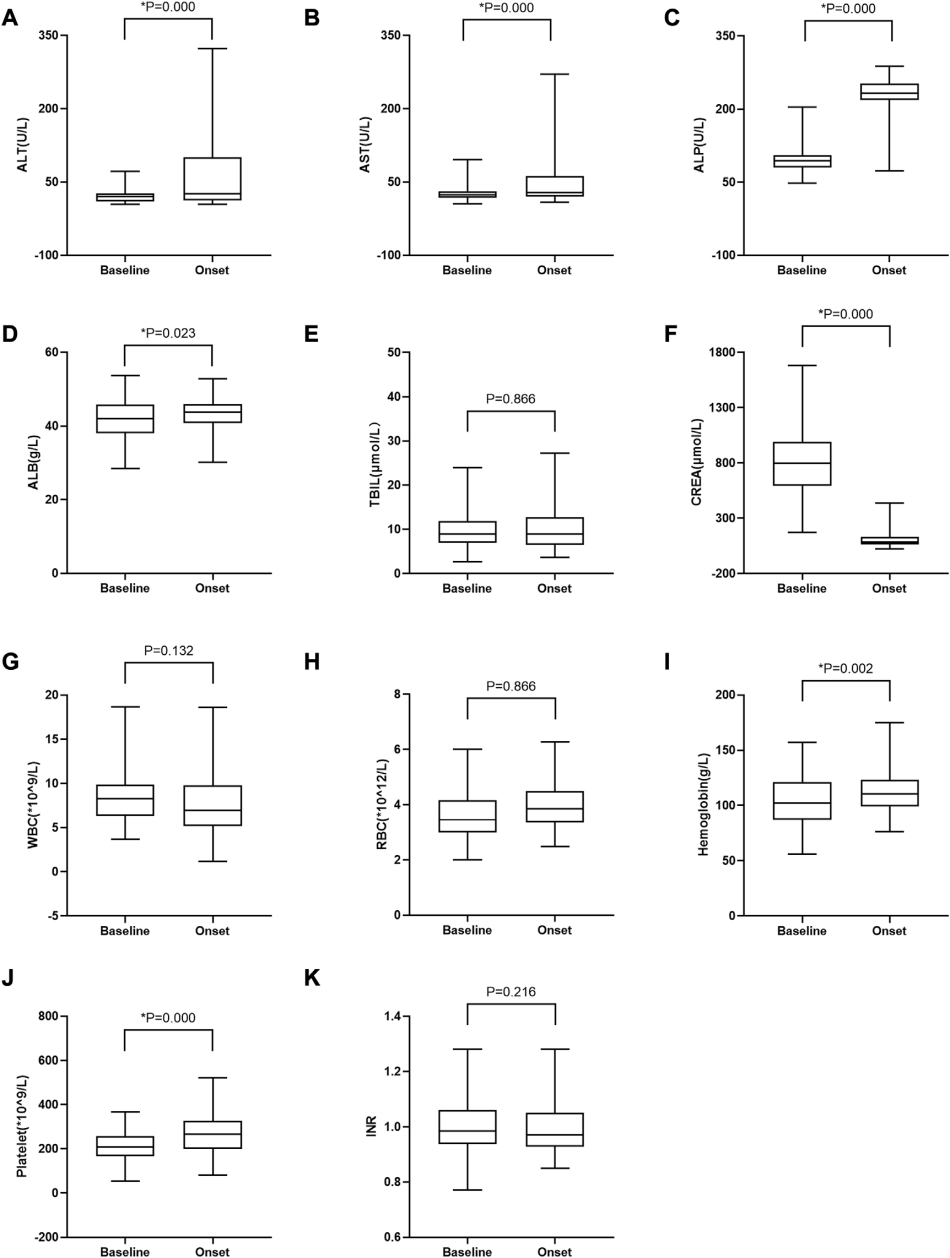
Changes in laboratory test values between baseline and at tac-DILI onset post-tacrolimus treatment in patients with tac-DILI (*n* = 90). **(A)** ALT; **(B)** AST; **(C)** ALP; **(D)** ALB; **(E)** TBIL; **(F)** CREA; **(G)** WBC; **(H)** RBC; **(I)** Hemoglobin; **(J)** Platelet; **(K)** INR. Horizontal bars represent the median value, boxes represent the interquartile range and whiskers indicate the minimum and maximum value. Wilcoxon’s test was used to compare laboratory test values between baseline and at tac-DILI onset post-tacrolimus treatment. *p<0.05. ALT: alanine aminotransferase; AST: aspartate aminotransferase; ALP: alkaline phosphatase; ALB: albumin; TBIL: total bilirubin; CREA: creatinine; WBC: white blood cell; RBC: red blood cell; INR: international normalized ratio.

To further investigate whether other body functions were affected by tacrolimus treatment in patients with tac-DILI, various biochemical parameters were compared before and after tacrolimus treatment. As shown in Figure 3, the median values of ALT, AST, ALP, RBC, ALB, hemoglobin, and platelets increased significantly following tacrolimus treatment, whereas those of CREA and WBC decreased significantly.

### 3.4 Risk factors for tac-DILI

**FIGURE 4:**
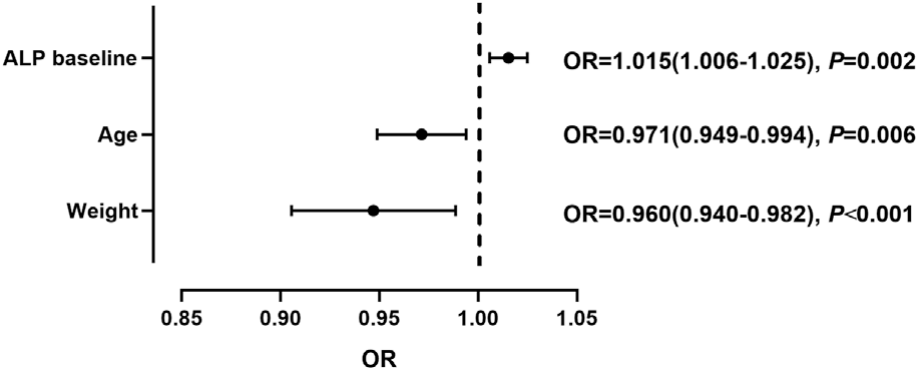
Independent risk factors for tacrolimus-induced liver injury.

90 patients with tac-DILI were matched with 360 non-tac-DILI patients; the characteristics of the tac-DILI and non-tac-DILI groups are presented in Table 2. The significant predictors of tac-DILI were subsequently introduced into the backward stepwise logistic regression model. Independent risk factors predicting tac-DILI were: ALP baseline (OR = 1.015, 95% CI = 1.006–1.025, *p* = 0.002), age (OR = 0.971, 95% CI = 0.949–0.994, *p* = 0.006), and weight (OR = 0.960, 95% CI = 0.940–0.982, *p* < 0.001) (Figure 4).

## 4 Discussion

Currently, data on tac-DILI are limited to reports from pre-marketing clinical trials and post marketing cases. To the best of our knowledge, this is the first study to describe the incidence, characteristics, prognosis, and risk factors of tac-DILI in renal transplant recipients. This study was supported by real-world data and included a relatively large sample size, which may provide a more reliable reference for the prevention and treatment of tac-DILI.

Administration of tacrolimus, cyclosporine A, and mTOR inhibitors may cause liver injury in patients following liver transplantation (Tischer and Fontana, 2014). A cohort study from the United States analyzed the incidence, clinical presentation, and outcomes of liver injury following liver transplantation; immunosuppressive agents, including azathioprine and tacrolimus, were regarded as inducers (Sembera et al., 2012). However, there is a lack of definite causality assessment conclusions for tac-DILI in this cohort study. Furthermore, liver transplantation itself is a risk factor for liver injury, which may be a confounding factor in identifying DILI (Zhenglu et al., 2007). Nevertheless, the diagnosis of DILI has always been difficult owing to the lack of diagnostic biomarkers and specific clinical features; therefore, physicians need to rely on the diagnosis by exclusion (EASL, 2019). Accordingly, the RUCAM scale was adopted in our study, which demonstrated the feasibility of identifying DILI (Teschke and Danan, 2016).

Triple immunosuppressive regimen including glucocorticoid, MMF and tacrolimus is used in the renal transplant recipients for the prevention of rejection. In our study, when the liver injury was suspected related to the immunosuppressant, tacrolimus dose was decreased or discontinued according to the concentration, and the recovery or alleviation of liver injury was observed, but the doses of glucocorticoid and MMF were maintained unchanged. Besides, more cases of liver injury were reported related to CNIs including tacrolimus in transplant recipients, as compared to low dose of glucocorticoid and MMF. Thus, we believed that tacrolimus may be a main factor causing the DILI. However, the co-administration of glucocorticoid and MMF may also potentially prompt the liver injury, and the result in this study may be better referenced for the transplant population.

In our study, the incidence of tac-DILI was 8.9%, which was in accordance with that described on the drug label (1%–10%, defined as common). Meanwhile, it is reported that tacrolimus therapy is associated with mild to moderate elevations in serum enzyme levels in 5%–10% of patients according to LiverTox (www.livertox.nih.gov) (LiverTox, 2012). However, no criteria for liver injury have been defined and no specific data support tac-DILI. The latency time of DILI varies from days to years (EASL, 2019). Even for the same drug, there was a difference in latency time among the different patterns of liver injury (Jiang et al., 2021). As observed in our study, the cholestatic pattern had the longest latency time, followed by the mixed and hepatocellular patterns. Furthermore, the latency time of abnormal liver enzymes in patients with DILI was significantly shorter than that of liver injury, which indicated that potentially idiosyncratic hepatotoxicity induced by tacrolimus possibly occurred prior to when the criteria for DILI were met. The reasons for this difference remain unclear but may be related to the mechanism of tacrolimus-induced hepatotoxicity, such as idiosyncratic metabolic or immunologic reactions (Ferjani et al., 2016; Hoofnagle and Bjornsson, 2019). As we could see in the results, there were only one case of moderate tac-DILI and 89 mild, and no moderate-severe, severe and fatal cases of tac-DILI, which was consistent with results shown on LiverTox. On the one hand, this may be attributed to the concomitant low elevated level of TBIL in patients with tac-DILI. On the other hand, this may be due to timely dose reduction and use of hepatoprotective agents.

Generally, the patterns may be related to prognosis. Although a study showed that there was no significant association between the type of liver injury and recovery time (Medina-Caliz et al., 2016), most studies suggest that cholestatic and mixed patterns of liver injury require a longer recovery time (Andrade et al., 2006; Bjornsson et al., 2007), which is consistent with the results of our study. Most patients with tac-DILI had a cholestatic pattern (75.6%); however, their recovery rate (26.5%) was significantly lower than that of patients with a hepatocellular (75.0%) and mixed (66.7%) patterns. Similarly, previous studies have also suggested that a prolonged disease course occurs more commonly in patients with cholestatic pattern (Chalasani et al., 2015). Most patients with tac-DILI have mild liver injury. On the one hand, this may be attributed to early detection and intervention, including withdrawal and dose reduction of tacrolimus and use of hepatoprotective agents. On the other hand, the liver injury caused by tacrolimus may be self-limiting (LiverTox, 2012).

Additionally, the present study is the first to report that tac-DILI tends to occur at a younger age. Drug use is recognized as a cause of pediatric liver disease, but little is known about DILI in children and adolescents (Ferrajolo et al., 2010; Ye et al., 2021). Age distribution analysis showed that 15.2% and 53.3% of non-tac-DILI and patients with tac-DILI, respectively, were ≤18 years of age. Owing to incomplete maturity of vital functions, children show significant differences in drug absorption, distribution, metabolism, and excretion compared to adults (Anderson, 2002), which may make children more susceptible to DILI. Low body weight is also an independent risk factor for tac-DILI. Physiological factors, such as body weight and organ volume, can affect drug clearance, which is associated with DILI. Moreover, allometric models that account for differences in body weight and age have been adopted to predict drug clearance (Mahmood, 2015; Shi et al., 2017).

The current study has some limitations. First, the study is susceptible to some bias because of the retrospective nature of the study and the results relied on the accuracy of the electronic medical records. Additionally, all the patients included in the study were from the post-renal transplantation population, which, while ensuring homogeneity and consistency in clinical practice, may reflect a specific case mix of post-renal transplantation instead of patients in other disease populations.

In conclusion, a relatively higher incidence of tac-DILI was found in renal transplant patients based on real-world data, and the most common liver injury type was of the cholestatic subtype. Young age, low body weight, and abnormal baseline ALP levels are independent risk factors for tac-DILI.

## Data Availability

The original contributions presented in the study are included in the article/supplementary material, further inquiries can be directed to the corresponding authors.

## Abbreviations

ALP, alkaline phosphatase; ALT, alanine aminotransferase; AST, aspartate aminotransferase; CNI, calcineurin inhibitors; CPG, clinical practice guidelines; C_0_, blood trough concentration; DILI, drug-induced liver injury; EASL, European association for the study of the liver; GGT, gamma-glutamyl transferase; KDIGO, Kidney Disease: Improving Global Outcomes; MMF, mycophenolate mofetil; RUCAM, Roussel Uclaf causality assessment method; TBIL, total bilirubin; tac-DILI, tacrolimus-induced liver injury.

## References

[B1] Anderson G. D. (2002). Children versus adults: Pharmacokinetic and adverse-effect differences. Epilepsia 43, 353–359. 10.1046/j.1528-1157.43.s.3.5.x 12060006

[B2] Andrade R. J. Lucena M. I. Kaplowitz N. Garcia-Munoz B. Borraz Y. Pachkoria K. (2006). Outcome of acute idiosyncratic drug-induced liver injury: Long-term follow-up in a hepatotoxicity registry. Hepatology 44 (6), 1581–1588. 10.1002/hep.21424 17133470

[B3] Bjornsson E. Kalaitzakis E. Av K. V. Alem N. Olsson R. (2007). Long-term follow-up of patients with mild to moderate drug-induced liver injury. Aliment. Pharmacol. Ther. 26 (1), 79–85. 10.1111/j.1365-2036.2007.03355.x 17555424

[B4] Bodell M. (2015). Immunosuppressive medications in kidney transplantation, Comprehensive clinical nephrology. St. Louis, Missouri: Saunders.

[B5] Chalasani N. Bonkovsky H. L. Fontana R. Lee W. Stolz A. Talwalkar J. (2015). Features and outcomes of 899 patients with drug-induced liver injury: The DILIN prospective study. Gastroenterology 148 (7), 1340–1352.e7. 10.1053/j.gastro.2015.03.006 25754159 PMC4446235

[B6] Corrigan-Curay J. Sacks L. Woodcock J. (2018). Real-world evidence and real-world data for evaluating drug safety and effectiveness. JAMA 320 (9), 867–868. 10.1001/jama.2018.10136 30105359

[B7] EASLClinical Practice Guideline Panel: ChairPanel membersEASL Governing Board representative (2019). EASL clinical practice guidelines: Drug-induced liver injury. J. Hepatol. 70 (6), 1222–1261. 10.1016/j.jhep.2019.02.014 30926241

[B8] Ferjani H. El Arem A. Bouraoui A. Achour A. Abid S. Bacha H. (2016). Protective effect of mycophenolate mofetil against nephrotoxicity and hepatotoxicity induced by tacrolimus in Wistar rats. J. Physiol. Biochem 72 (2), 133–144. 10.1007/s13105-015-0451-7 26746208

[B9] Ferrajolo C. Capuano A. Verhamme K. M. Schuemie M. Rossi F. Stricker B. H. (2010). Drug-induced hepatic injury in children: A case/non-case study of suspected adverse drug reactions in VigiBase. Br. J. Clin. Pharmacol. 70 (5), 721–728. 10.1111/j.1365-2125.2010.03754.x 21039766 PMC2997312

[B10] Gonwa T. Mendez R. Yang H. C. Weinstein S. Jensik S. Steinberg S. (2003). Randomized trial of tacrolimus in combination with sirolimus or mycophenolate mofetil in kidney transplantation: Results at 6 months. Transplantation 75 (8), 1213–1220. 10.1097/01.TP.0000062837.99400.60 12717205

[B11] Hoofnagle J. H. Bjornsson E. S. (2019). Drug-induced liver injury - types and phenotypes. N. Engl. J. Med. 381 (3), 264–273. 10.1056/NEJMra1816149 31314970

[B12] Jiang F. Yan H. Liang L. Du J. Jin S. Yang S. (2021). Incidence and risk factors of anti-tuberculosis drug induced liver injury (DILI): Large cohort study involving 4652 Chinese adult tuberculosis patients. Liver Int. 41 (7), 1565–1575. 10.1111/liv.14896 33866661

[B13] Kasiske B. L. Zeier M. G. Chapman J. R. Craig J. C. Ekberg H. Garvey C. A. (2010). KDIGO clinical practice guideline for the care of kidney transplant recipients: A summary. Kidney Int. 77 (4), 299–311. 10.1038/ki.2009.377 19847156

[B14] LiverTox (2012). Clinical and research information on drug- induced liver injury. Bethesda (MD): National Institute of Diabetes and Digestive and Kidney Diseases. 31643176

[B15] Mahmood I. (2015). Prediction of drug clearance in children: A review of different methodologies. Expert Opin. Drug Metab. Toxicol. 11 (4), 573–587. 10.1517/17425255.2015.1019463 25740388

[B16] Medina-Caliz I. Robles-Diaz M. Garcia-Munoz B. Stephens C. Ortega-Alonso A. Garcia-Cortes M. (2016). Definition and risk factors for chronicity following acute idiosyncratic drug-induced liver injury. J. Hepatol. 65 (3), 532–542. 10.1016/j.jhep.2016.05.003 27184533 PMC7458366

[B17] Mesar I. Kes P. Hudolin T. Basic-Jukic N. (2013). Rescue therapy with sirolimus in a renal transplant recipient with tacrolimus-induced hepatotoxicity. Ren. Fail. 35 (10), 1434–1435. 10.3109/0886022X.2013.828356 24028307

[B18] Min S. I. Ha J. Kang H. G. Ahn S. Park T. Park D. D. (2013). Conversion of twice-daily tacrolimus to once-daily tacrolimus formulation in stable pediatric kidney transplant recipients: Pharmacokinetics and efficacy. Am. J. Transpl. 13 (8), 2191–2197. 10.1111/ajt.12274 23734831

[B19] Nankivell B. J. Borrows R. J. Fung C. L. O'Connell P. J. Allen R. D. Chapman J. R. (2003). The natural history of chronic allograft nephropathy. N. Engl. J. Med. 349 (24), 2326–2333. 10.1056/NEJMoa020009 14668458

[B20] Pirsch J. D. Miller J. Deierhoi M. H. Vincenti F. Filo R. S. (1997). A comparison of tacrolimus (FK506) and cyclosporine for immunosuppression after cadaveric renal transplantation. FK506 Kidney Transplant Study Group. Transplantation 63 (7), 977–983. 10.1097/00007890-199704150-00013 9112351

[B21] Rodriguez-Rodriguez A. E. Porrini E. Hornum M. Donate-Correa J. Morales-Febles R. Khemlani R. S. (2021). Post-transplant diabetes mellitus and prediabetes in renal transplant recipients: An update. Nephron 145 (4), 317–329. 10.1159/000514288 33902027

[B22] Sembera S. Lammert C. Talwalkar J. A. Sanderson S. O. Poterucha J. J. Hay J. E. (2012). Frequency, clinical presentation, and outcomes of drug-induced liver injury after liver transplantation. Liver Transpl. 18 (7), 803–810. 10.1002/lt.23424 22389256 PMC3396746

[B23] Shi Q. Yang X. Greenhaw J. J. Salminen A. T. Russotti G. M. Salminen W. F. (2017). Drug-induced liver injury in children: Clinical observations, animal models, and regulatory status. Int. J. Toxicol. 36 (5), 365–379. 10.1177/1091581817721675 28820004

[B24] Silva H. J. Yang H. C. Abouljoud M. Kuo P. C. Wisemandle K. Bhattacharya P. (2007). One-year results with extended-release tacrolimus/MMF, tacrolimus/MMF and cyclosporine/MMF in de novo kidney transplant recipients. Am. J. Transpl. 7 (3), 595–608. 10.1111/j.1600-6143.2007.01661.x 17217442

[B25] Taniai N. Akimaru K. Ishikawa Y. Kanada T. Kakinuma D. Mizuguchi Y. (2008). Hepatotoxicity caused by both tacrolimus and cyclosporine after living donor liver transplantation. J. Nippon. Med. Sch. 75 (3), 187–191. 10.1272/jnms.75.187 18648179

[B26] Teschke R. Danan G. (2016). Diagnosis and management of drug-induced liver injury (DILI) in patients with pre-existing liver disease. Drug Saf. 39 (8), 729–744. 10.1007/s40264-016-0423-z 27091053

[B27] Tischer S. Fontana R. J. (2014). Drug-drug interactions with oral anti-HCV agents and idiosyncratic hepatotoxicity in the liver transplant setting. J. Hepatol. 60 (4), 872–884. 10.1016/j.jhep.2013.11.013 24280292 PMC4784678

[B28] Wijdicks E. F. (2001). Neurotoxicity of immunosuppressive drugs. Liver Transpl. 7 (11), 937–942. 10.1053/jlts.2001.27475 11699028

[B29] Ye L. Feng Z. Huang L. Guo C. Wu X. He L. (2021). Causality evaluation of drug-induced liver injury in newborns and children in the intensive care unit using the updated Roussel Uclaf causality assessment method. Front. Pharmacol. 12790108, 790108. 10.3389/fphar.2021.790108 PMC8721278 34987403

[B30] Zhenglu W. Hui L. Shuying Z. Wenjuan C. Zhongyang S. (2007). A clinical-pathological analysis of drug-induced hepatic injury after liver transplantation. Transpl. Proc. 39 (10), 3287–3291. 10.1016/j.transproceed.2007.08.096 18089373

